# Education, Political Party, and Football Viewership Predict Americans' Attention to News About Concussions in Sports

**DOI:** 10.3389/fspor.2021.655890

**Published:** 2021-05-26

**Authors:** Andrew M. Lindner, Daniel N. Hawkins

**Affiliations:** ^1^Department of Sociology, Skidmore College, Saratoga Springs, NY, United States; ^2^Department of Sociology and Anthropology, College of Arts and Sciences, University of Nebraska Omaha, Omaha, NE, United States

**Keywords:** concussions, sport, news attention, football, polarization

## Abstract

News outlets, sports coverage, and even Hollywood movies have highlighted the growing body of research documenting the long-term negative consequences of traumatic injury in athletics, particularly, (sports-related) concussions. Despite so much media coverage, little is known about how much attention members of the American public pay to sports concussion news. Disparities in attention to concussion news among sociodemographic groups may contribute to further inequalities in rates of concussions that stem from participation in collision sports. In this study, using a 2017 nationally representative survey of US residents (*n* = 964), we examine the social, political, and demographic correlates of individuals' attention to news about concussions in sports. Regression results indicate that older, more educated, Democratic-leaning respondents reported that they pay more attention to news about concussions. Additionally, respondents with a greater past competitive athletic participation and those who regularly watch baseball and football reported higher levels of attention to concussion news. These findings are consistent with previous research showing higher levels of news consumption and trust in science among the highly educated and Democrats. The increased levels among football viewers may be in response to the inclusion of concussion news in game coverage.

## Introduction

Over the past decade, the consequences of traumatic head injuries in sports have garnered a wave of news coverage (Ahmed and Hall, 2017; Bachynski, 2019). Bolstered by a growing body of biomedical evidence (Mez et al., 2017; Alosco et al., 2018; Binney and Bachynski, 2018; Alosco and Stern, 2019; Mackay et al., 2019), there have been regular headlines, magazine features, documentaries, and even a Hollywood movie about the long-term risks of concussions^1^ in athletes from children to professionals. The focus on sports-related concussions (SRC) in particular has overtaken concern about other types of sports injury, and this focus has simultaneously expanded from fighting sports to all collision and contact sports and beyond (Malcolm, 2020). The subsequent “concussion crisis” in sports is a cultural rather than “natural” phenomenon due to the contested understanding, meaning, and implications of concussions for participation in sports (Malcolm, 2020). So, we ask, how much attention has the American public paid in general to this important issue of safety in sports? In addition, which groups in particular are most likely to pay attention to news about concussions? Given the seriousness of the consequences of sports-related concussions, disparities in attention to news about concussions may exacerbate inequalities in participation in collision sports, and subsequently, the risk of traumatic sports injury.

Research shows that more highly-educated people tend to pay closer attention to news in general (Stroud, 2017), and are more trusting of science (Gauchat, 2012; Veldkamp et al., 2017). It is possible that highly-educated people's self-selected news media exposes them to more coverage of the negative consequences of concussions. Furthermore, when exposed to journalistic descriptions of scientific studies related to concussions, they may be more willing to accept the legitimacy of such findings. Qualitative studies and journalistic accounts have found that more educated and affluent parents are increasingly turning away from American football due to concerns about the long-term effects of concussions (Semuels, 2019; Whalen, 2019). While it seems likely that greater educational attainment is associated with paying more attention to news about concussions in sports, to date, that has not been established in quantitative research. Controlling for other factors, we hypothesize that more educated respondents will report higher levels of attention to news about concussions in sports.

As with educational attainment, in recent years, a number of scientific and medical issues have become polarized along political lines in the US. Members of the more conservative Republican Party are more likely to distrust scientific evidence than either independents (voters unaffiliated with either of the two dominant parties) or members of the left-leaning Democratic Party (Gauchat, 2012; Nisbet et al., 2015; Whitehead and Perry, 2020). However, such findings are complicated by emergent demographic cleavages in party composition. In particular, since the mid-2010s, having a college degree has become an increasingly strong predictor of supporting Democratic policies and candidates over those of the Republican Party (Abramowitz and McCoy, 2019). To the extent that Republicans pay less attention to concussion news, it is unclear whether that is an artifact of the lower levels of educational attainment among Republicans. Nonetheless, controlling for other factors, we hypothesize that for ideological reasons, Republicans will report lower levels of attention to news about concussions in sports than either independents or Democrats.

Finally, it is tempting to think that sports fans in general, and especially those who follow contact and collision sports with an elevated concussion risk, would be more attuned to the coverage of concussions. Some discussion of concussions is often a part of the game day coverage on TV. When players are held out of games due to concussion symptoms or attended to by medical staff after a significant head impact, it may increase interest in the latest findings on concussions in sports. However, it is equally possible that a greater attention to concussions among fans of a given sport is simply reflective of the underlying demographic composition of its fanbase. The sports people choose to watch, enroll their children in, and actively play are strongly associated with their social class, gender, race, education, politics, and region in the US (Martin, 2015; Allison and Barranco, 2020). Fans of the NBA, for example, tend to skew liberal and urban, while regular viewers of ice hockey tend to be white and from northern parts of the US (Kalman-Lamb, 2018; Lindner and Hawkins, 2021). Likewise, the choice to enroll a child in Pop Warner football, a pay-for-play club soccer team, or the local rec basketball league has to do with family finances and a sense of safety, but also cultural and ideological considerations (Boufous et al., 2007; Lindner and Hawkins, 2012; Strandbu et al., 2019; Whalen, 2019). Still, the risks associated with concussions are considerable in a number of “collision sports,” including American football and ice hockey, and to a lesser extent, in “contact sports” like basketball and soccer (Binney, 2019; Kerr et al., 2019). In this study, we measure the viewership of two collision sports (football and ice hockey) and three contact sports (soccer, basketball, and baseball). All things being equal, we would expect that sports viewers, particularly in collision sports with more frequent concussions, would pay a greater attention to concussion news than non-sports viewers.

American football deserves a special mention here. In news coverage of concussions in the United States, no sport is mentioned more frequently than tackle football—and with good reason. In the US, football accounts for far more of the concussions at the high school and college levels than other sports. When holding gender constant (i.e., comparing football to other men's sports), football has much higher rates of head injuries than other sports (Binney, 2019; Kerr et al., 2019). Despite these facts, prominent figures within football have gone to great lengths to suppress the coverage of concussions in the name of defending the sport (Fainaru-Wada and Fainaru, 2014; Bachynski, 2019). It is possible that regular football fans are “in denial” about concussions, and actively avoid news about concussions. On the other hand, given the strict and well-publicized concussion protocols that have been developed in the past decade, it has been difficult to watch the NFL or college football without being exposed to at least some coverage of concussions.

In this study, using data from a nationally representative survey, we examine the social, political, and demographic correlates of individuals' attention to news about concussions in sports. In doing so, we contribute to our understanding of how closely the American public is following these issues, and reveal which social sub-groups are attentive to this pressing health and safety problem. In particular, the findings of this research aim to disentangle the associations among educational attainment, political party, sports viewership, and attention to concussion news. These results may prove useful to advocates for safety in sports, considering which demographic groups to target with information about the risks of concussions.

## Method

### Sample and Missing Data

The data for this study were collected in October 2017 by the polling firm SurveyMonkey Audience using a nationally-representative sampling frame with original survey questions created by the authors. Of the 1,089 respondents who were contacted and agreed to participate, 1,017 (93.4%) completed the survey, with a median completion time of just under 6 min^2^. White and female respondents were overrepresented in the sample relative to the US population, so individual sample weights for race and gender were used to produce a representative sample for the regression analyses and creation of the figures. We limited the sample to the 964 respondents who had a valid response to the dependent variable. The measurement of all variables is outlined in the next section.

Missing data on the independent variables were minimal (<4%) with the exception of income (13%; see Table 1). For the regression analyses that follow, we used multiple imputation in Stata 15.1 (*mi impute* command) based on 10 data sets to maximize statistical power (*N* = 964). As a check on our missing data strategy, we also conducted regression analyses with listwise deletion (*N* = 766), which produced substantively nearly identical results (see Supplementary Table 1). Though the ordinal dependent variable violates the distributional assumptions of ordinary least squares (OLS) regression, OLS is generally robust to such violations. However, as a check, we conducted an ordinal logit analysis. The results, presented in the Supplementary Table 2, showed no changes in significance or magnitude. For the sake of parsimony and clarity, we describe only the OLS findings in the Results section.

**Table 1:** Descriptive statistics for all study variables (Unweighted).

	***M***	***SD***	***N***
Attention to concussion news	2.759	0.968	964
Education (High School or Less)	0.131	–	949
Education (Some College)	0.321	–	949
Education (Bachelor's Degree)	0.301	–	949
Education (Graduate Degree)	0.247	–	949
Republican	0.293	–	946
Democrat	0.415	–	946
Independent	0.292	–	946
Watch Baseball	2.271	1.184	955
Watch Basketball	1.935	0.989	962
Watch Soccer	1.649	0.958	934
Watch Hockey	1.761	1.040	954
Watch Football	2.476	1.138	964
Athlete Experience	2.588	1.395	957
Age (18–29)	0.214	–	950
Age (30–44)	0.258	–	950
Age (45–59)	0.265	–	950
Age (60+)	0.263	–	950
White	0.765	–	932
Female	0.536	–	950
Married	0.493	–	948
Income (< $25,000)	0.230	–	834
Income ($25,000–49,999)	0.200	–	834
Income ($50,000–74,999)	0.179	–	834
Income ($75,000–124,999)	0.218	–	834
Income ($125,000 or More)	0.173	–	834
Northeast	0.192	–	938
Midwest	0.225	–	938
West	0.242	–	938
South	0.341	–	938
Rural	0.202	–	950
Suburban	0.453	–	950
Urban	0.345	–	950

### Measures

The dependent variable asked the respondents, “How much attention have you paid to news coverage of concussions in sports over the past few years?” with response categories from 1 = *none*, 2 = *a little bit*, 3 = *some*, and 4 = *a great deal*.

The three primary independent variables were educational attainment, political party identification, and how often the respondents reported watching five major sports. Educational attainment was a set of dummy variables that assessed the highest degree the respondents had earned: *high school or less* (reference), *some college, bachelor's degree*, and *graduate degree*. Political party identification was a set of three dummy variables coded as *Republican* (reference group), *Democrat*, and *Independent*. *Watch [sport]* was a set of five ordinal variables that assessed how often respondents watched the major professional and/or collegiate leagues associated with five major sports when in-season (baseball, basketball, soccer, hockey, and football) on a scale from 1 = *never* to 5 = *rarely miss a game*.

We controlled for a number of other sociodemographic characteristics, which may plausibly be associated with attention to concussion news as well as the three primary predictors. *Age* was a set of dummy variables coded as *18–29, 30–44* (reference), *45–59*, and *60 or older*. Race was coded as 1 = *White* and 0 = non-White. Gender and marital status were binary variables, coded respectively as 1 = *female* and 0 = male and 1 = *married* and 0 = not married. Annual *income* was a set of dummy variables divided roughly by quintiles (see Table 1): < *$25,000, $25,000–49,999, $50,000–74,999* (reference), *$75,000–124,999*, and *$125,000 or more*. Geographic region was a set of four dummy variables consisting of *South* (reference group), *Northeast, Midwest*, and *West*. Community type was a set of three dummy variables that includes *urban* (reference group), *suburban*, and *rural*. Finally, *athlete experience* was an ordinal variable that assessed the highest level of competitive sports respondents ever played in, coded as 1 = *never participated*, 2 = *youth recreational league*, 3 = *high school freshman or junior varsity*, 4 = *high school varsity*, and 5 = *collegiate and/or professional*.

### Analytic Strategy

We first calculate the means or proportions and standard deviations for attention to concussion news and the independent variables. We then estimated an ordinary least squares (OLS) regression, which included all the independent and control variables. To test for potential issues with multicollinearity, we ran the Variance Inflation Factors (VIF) using the *collin* Stata package. All VIFs were below 2.5, suggesting no indication of multicollinearity in our model. To underscore the magnitudes of associations between the primary predictors and the dependent variable, we produced three bar charts visualizing cross-tabulations. The descriptive statistics are unweighted while the regression analysis and figures utilize weighted data.

## Results

The majority of the respondents reported that they are paying “some” (39%) or “a great deal” (25%) of attention to news of concussions in sports, with only 25% saying “a little bit” and 11% saying “none.” Overall, these results indicated a fairly high level of attention to concussion news.

**Figure 1:**
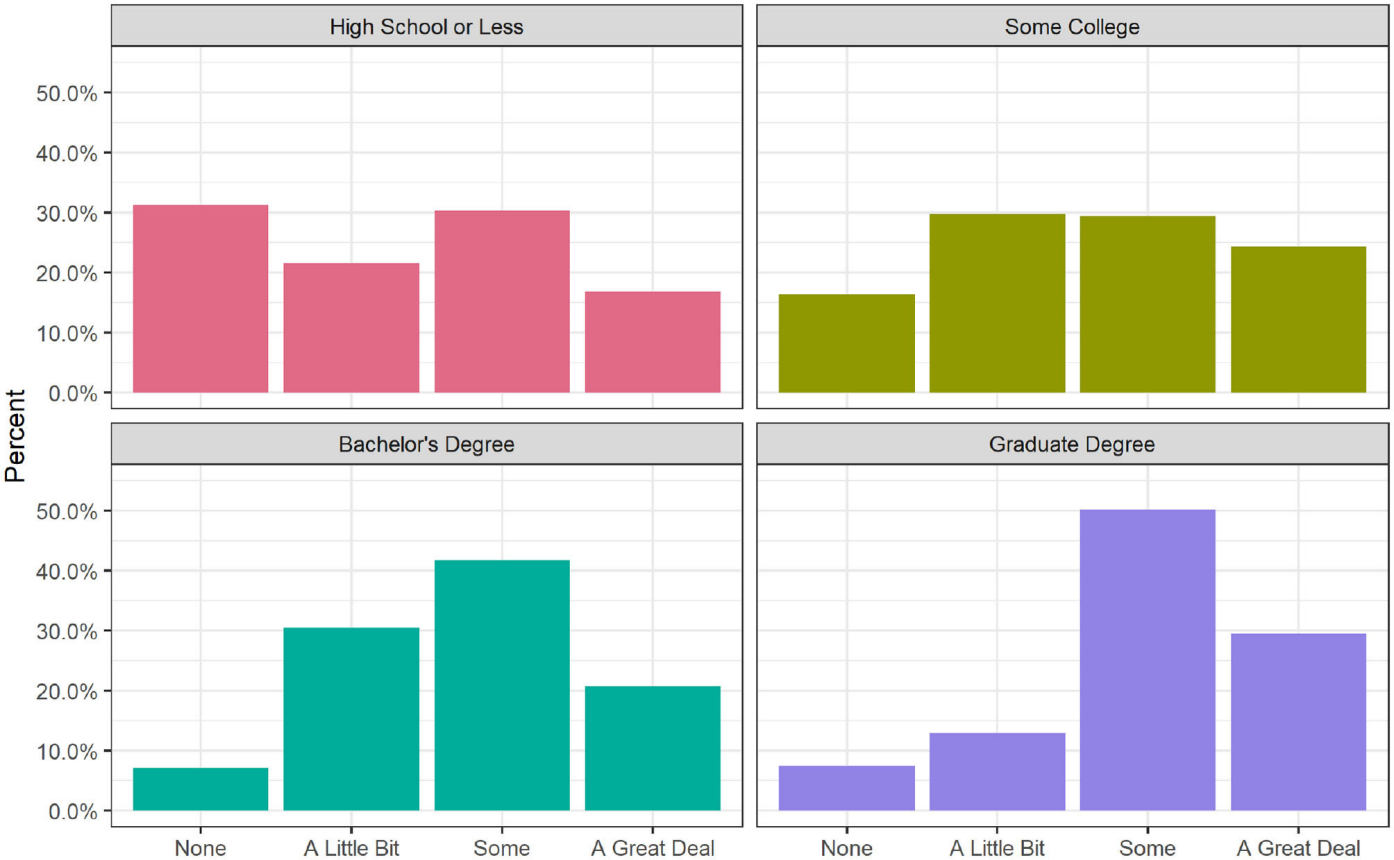
Attention to concussion news by educational attainment (Weighted).

**Figure 2:**
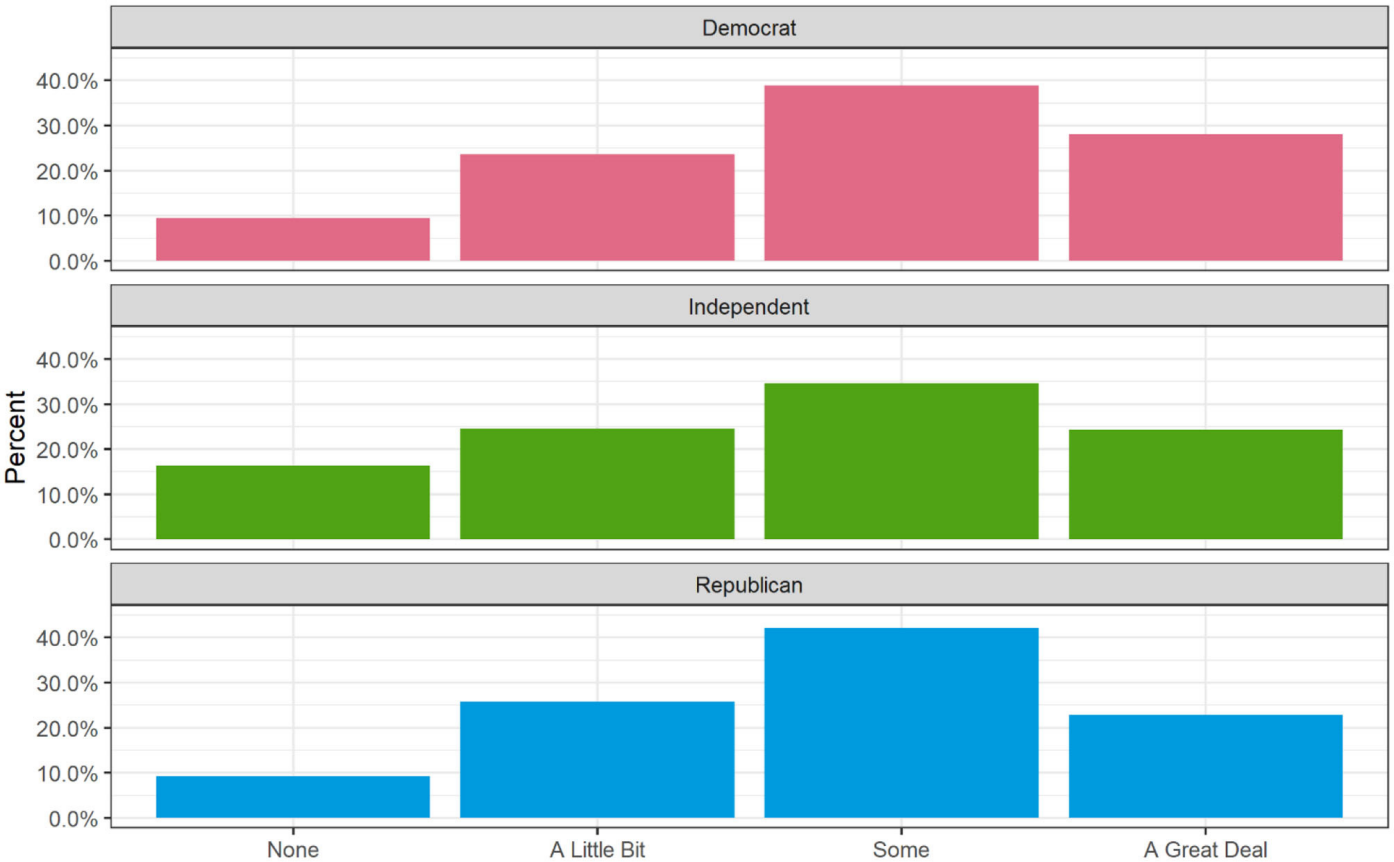
Attention to concussion news by political party (Weighted).

**Figure 3:**
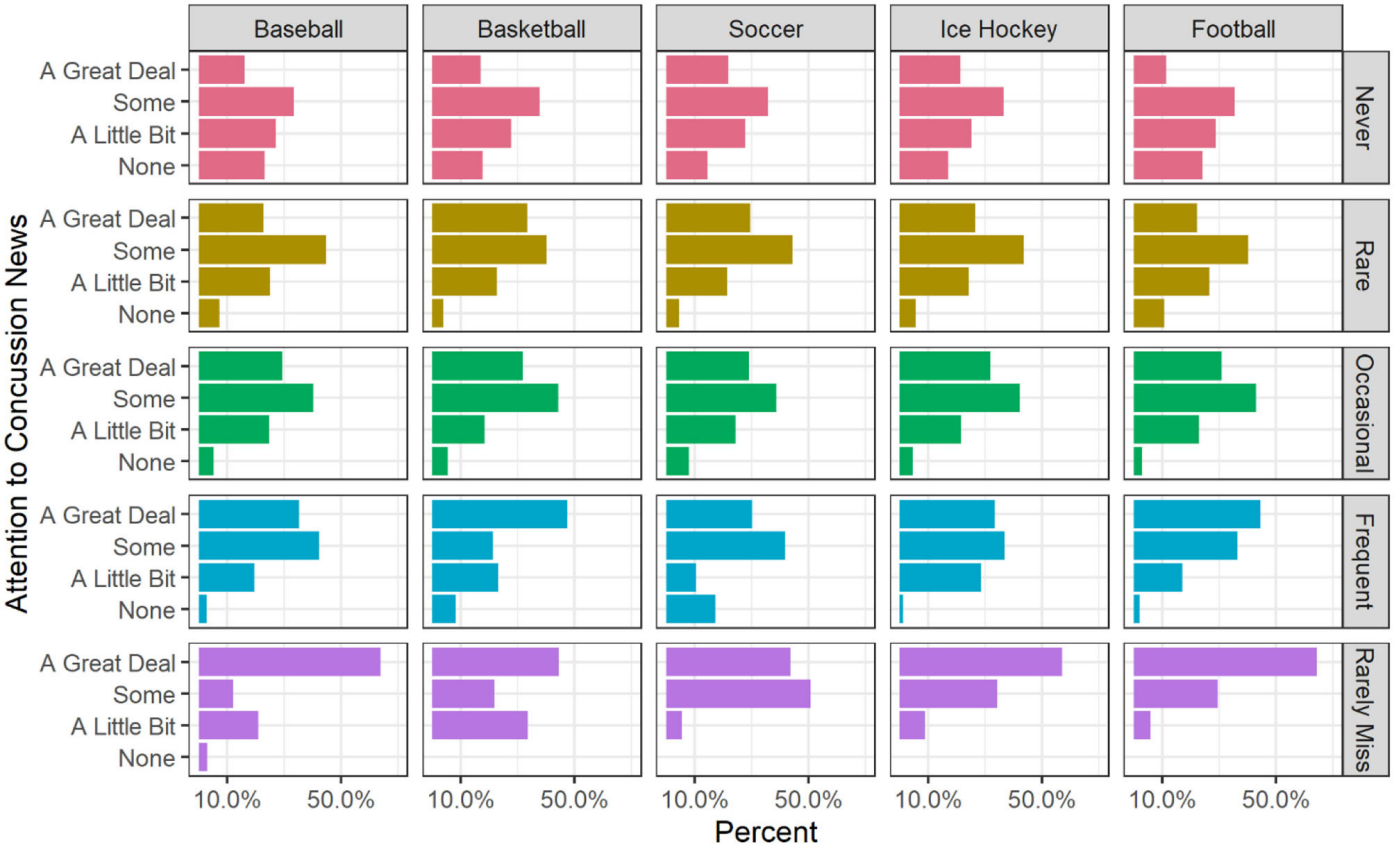
Attention to concussion news by frequency of watching sports (Weighted).

Figures 1–3 depict the bivariate relationships between each of the hypothesized independent variables and attention to concussion news. As seen in Figure 1, about 30% of those with graduate degrees, 21% of bachelor's holders, and 24% of those with some college are paying a great deal of attention to concussion news as compared to only 17% of respondents with a high school diploma or less. Rather than being a clear linear increase for each additional level of education, the difference between high school or less and at least some college seems to be an important dividing line in attention to concussion news, with another jump up in attention paid for those holding graduate degrees. Figure 2 reveals a fairly weak relationship between political party and attention concussion news. Democrats report paying the most attention, with 28% indicating that they are paying a great deal of attention and only 9% paying no attention. The proportions for Republicans are not radically different (23% report paying a great deal of attention and 9% report paying no attention). The most notable group difference is that 16% of independents report having paid no attention to news of concussions in sports.

Figure 3 depicts the relationship between the frequency of watching a sport (from “Never” to “Rarely Miss a Game”) and attention to concussion news for each of the five sports. Across all five sports, those who watch the sport frequently or rarely miss a game report paying a great deal of attention to concussion news at higher rates than those who watch sports rarely or never. The pattern is most pronounced for baseball, ice hockey, and football where majorities of those who rarely miss a game are paying a great deal of attention (64, 57, and 65%, respectively).

Turning to the regression results (Table 2), we expected that, net of other characteristics, respondents with higher levels of education would report higher levels of attention to news about concussions in sports. We found support for this hypothesis. As compared to those with a high school diploma or less, respondents with some college, a bachelor's degree, or a graduate degree were about a quarter of a point higher on the 4-point scale of attention to concussions (*p* < 0.001).

**Table 2:** Regression model predicting attention paid to news of concussions in sports (Weighted).

	**Attention to concussion news**	
	***b***	***SE***
Education (High School or Less)	–	
Education (Some College)	0.267^**^	0.090
Education (Bachelor's Degree)	0.210*	0.094
Education (Graduate Degree)	0.288^**^	0.100
Republican	–	
Democrat	0.162^*^	0.067
Independent	0.062	0.072
Watch Baseball	0.059^*^	0.028
Watch Basketball	0.017	0.037
Watch Soccer	0.038	0.033
Watch Hockey	0.029	0.030
Watch Football	0.198^***^	0.032
Athlete Experience	0.066^**^	0.022
Age (18–29)	−0.344^***^	0.084
Age (30–44)	–	
Age (45–59)	0.306^***^	0.078
Age (60+)	0.425^***^	0.078
White	−0.046	0.074
Female	−0.032	0.057
Married	−0.083	0.062
Income (< $25,000)	−0.205	0.108
Income ($25,000–49,999)	−0.030	0.099
Income ($50,000–74,999)	–	
Income ($75,000–124,999)	0.031	0.093
Income ($125,000 or More)	0.034	0.103
Northeast	0.024	0.080
Midwest	−0.104	0.074
West	−0.092	0.074
Rural	0.052	0.079
Suburban	0.072	0.064
Constant	1.526^***^	0.175
*R*^2^	0.295	

*N = 964;*

* *p < 0.05,*

** *p < 0.01,*

*** *p < 0.001*.

We also hypothesized that Republicans would report lower levels of attention to concussion news. We found partial support for this hypothesis. While Democrats reported a significantly higher attention to concussion news than Republicans (*p* < 0.05), there was no significant difference between independents and Republicans. Moreover, the magnitude of the effect was relatively modest: Democrats were 0.162 higher than Republicans on a 4-point scale of attention to concussion news, when controlling for other factors.

In terms of sports, we expected that those who watched more sports, especially collision sports such as football and ice hockey, would report paying more attention to concussion news. In fact, watching hockey, basketball, or soccer had no significant effect on attention to concussions, controlling for the level of viewership of all five sports. However, those who watch more hours of football reported significantly higher levels of attention to concussion news (*p* < 0.001) as did those who watch more baseball (*p* < 0.05). However, the magnitudes of these two sports effects were quite different. The difference between someone who never watches baseball and a respondent who watches the maximum category of baseball is about a quarter of a point on a 4-point scale of attention to concussion news. In contrast, the difference between a respondent who never watches football and one who rarely misses a game was 0.792 on the 4-point scale. Those who watch football more often paid substantially more attention to news about concussions.

Among the control variables, there were some noteworthy results. First, several variables, including race, gender, marital status, income, region, and community type, were not significantly associated with attention to concussion news. Second, as compared to respondents in the 30–44-year-old age cohort, 18–29-year-olds report significantly lower levels of attention to concussion news (*p* < 0.001). Compared to the 30–44-year-olds, those in the 45–59-year-old age cohort reported 0.306 higher attention (*p* < 0.001), and the 60+ age cohort reported 0.425 higher attention to concussion news (*p* < 0.001).

There was also a statistically significant, if modest, effect of competitive athletic participation on attention to concussion news (*p* < 0.01). Those with more athletic experience reported higher levels of attention to news of concussions in sports. However, for example, the difference between someone whose highest level of athletic participation was a youth recreational league and someone who had played varsity in high school was only 0.132 on a 4-point scale.

## Discussion

Given the high degree of media coverage that concussions in sports have received in the recent years, it is perhaps unsurprising that over half of Americans report paying “some” or “a great deal” of attention to concussion news. The results of this study also showed that older and more educated Americans, Democrats, former athletes, and regular viewers of baseball and, especially, football have been paying more attention to these issues. Equally important, net of other factors, we did not observe differences by race, gender, income, marital status, region, or community type.

With a cross-sectional data, like ours, it is impossible to know the causal order or the precise mechanisms at work in these associations. However, we speculate that there may be different mechanisms behind the demographic effects and the sports viewership effects. Past research indicates that those who have higher levels of education and older Americans consume more news overall, including higher cultural capital forms of journalism, such as science news (Stroud, 2017). Democrats and people who have higher levels of education are also more likely to trust science and may seek out the latest findings in medicine, including those related to sports (Gauchat, 2012). It may be that older people, individuals who have higher levels of education, and Democrats tend to consume more of the types of news sources (e.g., public radio, longform journalism, elite print magazines) that offer more coverage of the emerging science regarding concussions in sports. Alternatively, the increased levels of attention to concussion news among people over 45 may be reflective of the fact that many people in those age cohorts have children and grandchildren playing collision sports. Since we did not directly measure whether the respondents were parents and if their children play collision sports, it is impossible to know from these data. Regardless, developmental scholars have long noted that a concern for guiding and shaping the next generation tends to develop in the middle age (Erikson and Erikson, 1997), which may include a desire to protect young people from the short- and long-term effects of concussions.

In terms of sports viewership, a possible mechanism accounting for the higher levels of attention may simply be that concussion news is now a standard and an unavoidable part of a game day coverage. Our study does not address the quality or accuracy of this coverage, which can vary widely depending on the training and motivation of the announcer or reporter (Concussion Legacy Foundation, 2021). While television segments like ESPN's “Jacked Up,” which openly glorified violent hits in NFL games (Gordon, 2013), are no longer a part of the mainstream culture, there is still media coverage that condones, if not celebrates, collisions that clearly put athletes at risk of sports-related concussions (Concussion Legacy Foundation, 2021). Furthermore, our measure of attention to concussion news did not ask whether this attention was voluntary or involuntary, welcome or unwelcome. It is easy to imagine a football fan with a defensive response to concussion news who, nonetheless, sits through it because it is a brief tangent before the commentators return to discussing passing and coverage schemes. In addition, viewers in certain social contexts, such as locals that deeply value American football and in states that have not created policy governing return-to-play following concussions, may be more likely to dismiss the seriousness or relevance of this coverage (Rotolo and Lengefeld, 2020). However, if we are observing a game day coverage effect, it is surprising that more frequent viewers of ice hockey and soccer, each with their fair share of concussions, did not report significantly higher rates of attention.

For those eager to increase awareness of the risks of concussions in sports, the lack of gender, racial, and income divisions in the self-reported attention to concussion news is heartening. Women and men, Whites and people of color, and higher or lower earners may be equally interested and receptive to the latest science on concussions, net of other social factors. The results regarding gender may be particularly surprising given that the propensity of men to adhere to the “sport ethic” and traditional norms of masculinity, often purposely avoiding this coverage because they are resistant to change their behavior in the face of concussion symptoms (Liston et al., 2018). In contrast, the education gap creates the possibility of inequalities in child safety, if college-educated parents are more closely attuned to the risks posed by traumatic head injuries. Similarly, the low levels of attention to concussion news among 18–29-year-olds—the exact group of adults who are mostly likely to be engaged in recreational or competitive collision sports—is troubling. These results suggest that advocates for raising awareness about concussions would do well to target informational campaigns at adults under 30 and non-college educated people. Of course, in a post-truth and politically divided society in which emotion often matters more than fact in shaping attitudes about public health, attention to news about concussions does not necessarily indicate a willingness to make behavioral changes surrounding our treatment of them (Malcolm, 2020). Still, our study points to the social characteristics of people who are more likely to be aware of the concussion crisis and may be willing to at least engage in the debate about the prevalence and severity of sports-related concussion, including the long-term risk of CTE. Our survey data offer a correlational snapshot from one time point in the US with a limited number of variables. To better understand sociodemographic divisions, future researchers ought to ask a range of questions about the respondents' children and their potential sports involvement, as well as the respondents' own current participation in a sport as a player or coach. A wider set of questions about the preferred news sources might allow future research to better pinpoint which types of coverage spark interest in or successfully convey information about concussions in sports, with explicit attention to the messages that consumers receive and take away from this coverage (McGlynn et al., 2020). Knowledge about concussions should be examined in other countries to determine whether cultural, social, and political contexts play a role in this aspect of the concussion crisis (Malcolm, 2020). Finally, from a methodological standpoint, content analyses that examine the quantity and quality of discussion of concussions during game coverage across various sports would be valuable.

Despite these limitations, this study offers an initial opportunity to understand how the widespread coverage of concussions in sports has registered with the American public and teases apart the contribution of various overlapping social factors to concussion news awareness. While a majority of Americans are paying attention to the issue, the risk of concussions continues to be a serious problem for participants of all ages in collision sports. Our results show that individuals who are older, have higher levels of education, Democratic-leaning, and who watch football are especially likely to be paying attention to concussions in sports. These findings ought to direct the attention of sports safety advocates to engaging non-college educated people, young adults, fans of other collision sports, and people of all political stripes.

## Data Availability Statement

The datasets presented in this study can be found in online repositories. The names of the repository/repositories and accession number(s) can be found below: https://osf.io/k8bx5/?view_only=4960298d078e4ded893203b0dbf4456a.

## Ethics Statement

The studies involving human participants were reviewed and approved by Skidmore College Institutional Review Board. The patients/participants provided their written informed consent to participate in this study.

## Author Contributions

AL and DH collected the data, designed the research, conducted the analyses, and wrote the paper. Both authors contributed to the article and approved the submitted version.

## Conflict of Interest

The authors declare that the research was conducted in the absence of any commercial or financial relationships that could be construed as a potential conflict of interest.

## Footnotes

^1^Some researchers oppose the use of the term “concussion.” Neurologists Sharp and Jenkins (2015:172) went as far as to say, “It is time to stop using the term concussion as it has no clear definition and no pathological meaning.” Instead, they recommend “traumatic brain injury.” While we, as mere sociologists, have no stake to claim in this medical debate, “concussion” is the most frequently used word in media coverage (Ahmed and Hall, 2017) and, in all likelihood, by laypeople. Since we are describing sociological phenomena related to concussions, we adopt the terminology of media workers and the public, though it may be medically imprecise.

^2^SurveyMonkey Audience has an online panel of 80 million people in more than a hundred countries who have already submitted responses regarding basic demographics (e.g., gender, age, race, income, geographic location). All panelists must have Internet access, but may complete the surveys on a smartphone, tablet, or computer. For this survey, SurveyMonkey contacted non-institutionalized U.S. residents 18 and older with demographics balanced to the US Census proportions. For each survey panel the respondents complete, they earn credits, which can be exchanged for a contribution to one of several charitable organizations.
