# Supplementary material for: Education, Political Party, and Football Viewership Predict Americans' Attention to News About Concussions in Sports
Source: Front Sports Act Living. 2021 May 26;3:655890. doi: 10.3389/fspor.2021.655890 (PMC8187577; doi:10.3389/fspor.2021.655890)
Supplement: Supplementary file 1 [file Table_1.DOCX]

Supplementary Material

# Supplementary Tables

Table 1. OLS Regression Model Predicting Attention Paid to News of Concussions in Sports (Weighted)

|  | Attention to Concussion News | |
| --- | --- | --- |
|  | *b* | *SE* |
| Education (High School or Less) | -- |  |
| Education (Some College) | .312** | .100 |
| Education (Bachelor’s Degree) | .213* | .103 |
| Education (Graduate Degree) | .320** | .110 |
| Republican | -- |  |
| Democrat | .155* | .074 |
| Independent | .098 | .079 |
| Watch Baseball | .051 | .031 |
| Watch Basketball | .025 | .042 |
| Watch Soccer | .032 | .035 |
| Watch Hockey | .028 | .033 |
| Watch Football | .211*** | .036 |
| Athlete Experience | .054* | .024 |
| Age (18-29) | -.372*** | .090 |
| Age (30-44) | -- |  |
| Age (45-59) | .186* | .085 |
| Age (60+) | .384*** | .086 |
| White | -.064 | .081 |
| Female | -.000 | .063 |
| Married | -.044 | .067 |
| Income (Less than $25,000) | -.207* | .105 |
| Income ($25,000-49,999) | -.030 | .097 |
| Income ($50,000-74,999) | -- |  |
| Income ($75,000-124,999) | .048 | .094 |
| Income ($125,000 or More) | .074 | .104 |
| Northeast | .024 | .088 |
| Midwest | -.114 | .081 |
| West | -.089 | .081 |
| Rural | .060 | .089 |
| Suburban | .044 | .070 |
| Constant | 1.558*** | .185 |
| R^2^ | .300 |  |

*N*=766; List-wise deletion; * p<.05, ** p<.01, *** p<.001

Table 2. Ordered Logit Model Predicting Attention Paid to News of Concussions in Sports (Weighted)

|  | *Log Odds* | *SE* |
| --- | --- | --- |
| Education (High School or Less) | -- |  |
| Education (Some College) | 0.675** | 0.212 |
| Education (Bachelor’s Degree) | 0.511* | 0.217 |
| Education (Graduate Degree) | 0.690** | 0.233 |
| Republican | -- |  |
| Democrat | 0.375* | 0.155 |
| Independent | 0.159 | 0.165 |
| Watch Baseball | 0.121 | 0.065 |
| Watch Basketball | 0.043 | 0.086 |
| Watch Soccer | 0.086 | 0.075 |
| Watch Hockey | 0.064 | 0.069 |
| Watch Football | 0.466*** | 0.075 |
| Athlete Experience | 0.146** | 0.050 |
| Age (18-29) | -0.764*** | 0.192 |
| Age (30-44) | -- |  |
| Age (45-59) | 0.701*** | 0.182 |
| Age (60+) | 0.970*** | 0.182 |
| White | -0.132 | 0.176 |
| Female | -0.060 | 0.132 |
| Married | -0.215 | 0.142 |
| Income (Less than $25,000) | -0.468 | 0.249 |
| Income ($25,000-49,999) | -0.034 | 0.225 |
| Income ($50,000-74,999) | -- |  |
| Income ($75,000-124,999) | 0.045 | 0.210 |
| Income ($125,000 or More) | 0.121 | 0.238 |
| Northeast | 0.058 | 0.183 |
| Midwest | -0.235 | 0.172 |
| West | -0.202 | 0.168 |
| Rural | 0.093 | 0.182 |
| Suburban | 0.139 | 0.149 |
| Cut Point 1 | 0.385 | 0.414 |
| Cut Point 2 | 2.200*** | 0.415 |
| Cut Point 3 | 4.253*** | 0.430 |
| Pseudo R^2^ | 0.139 |  |

N=964; Multiple imputation on missing data; * p<.05, ** p<.01, *** p<.001
